# Normobaric oxygen may correct chronic cerebral ischemia‐mediated EEG anomalies

**DOI:** 10.1111/cns.13703

**Published:** 2021-07-09

**Authors:** Jia‐Yue Ding, Yu Liu, Gary‐B. Rajah, Zhi‐Ying Chen, Shi‐Yong Zhang, Yu‐Chuan Ding, Xun‐Ming Ji, Ran Meng

**Affiliations:** ^1^ Department of Neurology Xuanwu Hospital Capital Medical University Beijing China; ^2^ Department of Neurology Tianjin Medical University General Hospital Tianjin China; ^3^ Epilepsy Center Beijing Fengtai You’anmen Hospital Beijing China; ^4^ Department of Neurosurgery Jacobs School of Medicine and Biomedical Sciences University at Buffalo Buffalo NY USA; ^5^ Advanced Center of Stroke Beijing Institute for Brain Disorders Beijing China; ^6^ Department of China‐America Institute of Neuroscience Xuanwu Hospital Capital Medical University Beijing China; ^7^ Department of Interventional Neurology Beijing Fengtai You’anmen Hospital Beijing China; ^8^ Department of Neurosurgery Wayne State University School of Medicine Detroit MI USA; ^9^ Department of Neurosurgery Xuanwu Hospital Capital Medical University Beijing China

**Keywords:** brain dysfunction, chronic ischemia, electrophysiology, oxygen

## Abstract

**Aims:**

To explore the safety and efficacy of normobaric oxygen (NBO) on correcting chronic cerebral ischemia (CCI) and related EEG anomalies.

**Methods:**

This prospective randomized trial (NCT03745092) enrolled 50 cases of CCI patients, which were divided into NBO (8 L/min of oxygen supplement) group and control group (room air) randomly, and also enrolled 21 healthy volunteers. Two times of 30‐min EEG recordings with the interval of 45min of NBO or room air were analyzed quantitatively.

**Results:**

The CCI‐mediated EEG presented with two patterns of electrical activities: high‐power oscillations (high‐power EEG, *n* = 26) and paroxysmal slow activities under the normal‐power background (normal‐power EEG, *n* = 24). The fronto‐central absolute power (AP) of the beta, alpha, theta, and delta in the high‐power EEG was higher than that in healthy EEG (*p* < 0.05). The fronto‐central theta/alpha, delta/alpha and (delta + theta)/(alpha + beta) ratios in the normal‐power EEG were higher than those in healthy EEG (*p* < 0.05). The high‐power EEG in NBO group had higher fronto‐central AP reduction rates than those in control group (*p* < 0.05). NBO remarkably reduced the fronto‐central theta/alpha, delta/alpha, and (delta + theta)/(alpha + beta) ratios in the normal‐power EEG (*p* < 0.05).

**Conclusions:**

NBO rapidly ameliorates CCI‐mediated EEG anomalies, including attenuation of the abnormal high‐power oscillations and the paroxysmal slow activities associated with CCI.

## Graphical abstract

We provided clinical evidences for use of normobaric oxygen (NBO) in patients with chronic cerebral ischemia (CCI). NBO can rapidly ameliorate CCI‐related EEG anomalies, including attenuating the abnormal high‐power oscillations and suppressing the paroxysmal slow activities in the CCI condition. Long‐term NBO performance may still benefit in the CCI patients.

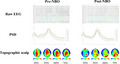

## 1 INTRODUCTION

As we all know, the chronic cerebral ischemia (CCI) is very popular and intractable condition in clinical setting. Patients with CCI often complain about long‐term non‐specific symptoms, such as headache, dizziness, memory decline, and insomnia, which seriously affected their quality of life.^1^ The predominant etiology of CCI is atherosclerotic stenosis, especially intracranial arterial stenosis (ICAS) in Chinese populations, which may be responsible for 33%–50% of ischemic stroke events and more than 50% transient ischemic attack (TIA).^2^, ^3^ The latest epidemiological statistics reported that CCI carries a high incidence in elderly people, occurring in more than two‐thirds of individuals older than 65 years of age.^1^ Despite statins and antiplatelet use can largely block stroke occurrence and recurrence induced by CCI, to date there are no effective strategies aimed at correcting CCI‐mediated brain dysfunctions.

Normobaric oxygen (NBO), supplied by nasal cannula or facemask with one atmosphere pressure (1 ATA = 101.325 kPa), has been utilized as an adjuvant therapy for correcting various diseases.^4^, ^5^, ^6^, ^7^ NBO could ameliorate hypoxia of brain tissue, correct brain dysfunction rapidly, and finally improve clinical outcomes.^8^, ^9^, ^10^, ^11^, ^12^, ^13^ However, to our knowledge, few studies about NBO on correcting CCI‐related brain dysfunctions are reported at present. EEG can comprehensively reflect the changes of brain function induced by cerebral ischemia and hypoxia; herein, we aimed to evaluate the efficacy of NBO on correcting CCI‐mediated brain dysfunctions by real‐time quantitative EEG analysis.

## 2 METHODS

### 2.1 Study design and participants

This is a proof‐of‐concept, assessor‐blinded, randomized controlled clinical trial registered on ClinicalTrials.gov (http://www.clinicaltrials.gov; Unique identifier: NCT03745092) and has been approved by the Institutional Ethics Committee of Xuanwu Hospital, Capital Medical University (Beijing, China) in accordance with the guidelines of the 1964 Declaration of Helsinki. Informed consent was obtained from all individuals prior to their enrollment. As the EEG features for CCI are currently not clearly defined, this study analyzed the abnormal EEG oscillations in CCI (Part I) for the first time, and explored the efficacy of NBO on correcting the EEG anomalies (Part II).

From December 2018 through December 2019, patients with imaging confirmed CCI induced by ICAS and/or extracranial carotid arterial stenosis (ECAS) at Xuanwu Hospital, Capital Medical University, were enrolled in this study and randomly entered into NBO group or control group according to the random number. The diagnosis of ICAS and ECAS was confirmed by magnetic resonance angiography (MRA) and/or computed tomographic angiography (CTA). Perfusion weighted imaging (PWI) scan was used to confirm the status of CCI, and the parameter used in this study was mainly the time‐to‐peak time (TTP). Prolonged TTP in territory of stenosis arteries was considered as hypo‐perfusion. All of the enrolled patients matched the following criteria.

Inclusion criteria: (1) age from 18 to 80 years; (2) ICAS and/or ECAS confirmed by imaging, with prolonged TTP; (3) NIHSS ≤3 and mRS ≤2; (4) signed the informed consent.

Exclusion criteria: (1) brain infarction occurrence within two months; (2) intracranial arterial aneurysm, dissection or malformation; (3) history of cerebral hemorrhage or subarachnoid hemorrhage; (4) brain trauma; (5) other brain injury or disorders; (6) austere diseases such as cancer, heart failure, and respiratory failures; (7) respiratory diseases; (8) severe liver and kidney dysfunction; (9) poor compliance.

In order to elucidate EEG features of CCI, a cohort of healthy volunteers was also incorporated into this study. This population was confirmed to harbor no cerebral vascular diseases and had no arterial atherosclerosis‐related risk factors (such as hypertension, diabetes, dyslipidemia, etc.).

### 2.2 Study procedures

In Part I, all of the enrolled patients was labeled as the CCI group and underwent 30‐min EEG recordings twice. During the interval of the two times of EEG recordings, the patients were randomly assigned to receive NBO (8 L/min of oxygen supplement for 45 min via simple mask) or room air (resting like lying, sitting, or walking at room air for 45 min without any other specific interventions) randomly according to the random number, and these two arms were labeled as NBO group and control group, respectively in Part II. All of the healthy volunteers were labeled as the health group and underwent 30‐min EEG recording once. All patients and volunteers were kept awake during EEG recording to eliminate twilight and sleep state confounding of EEG samples. The EEG analysts were blind to the clinical database and the specific interventions in this part.

**FIGURE 1:**
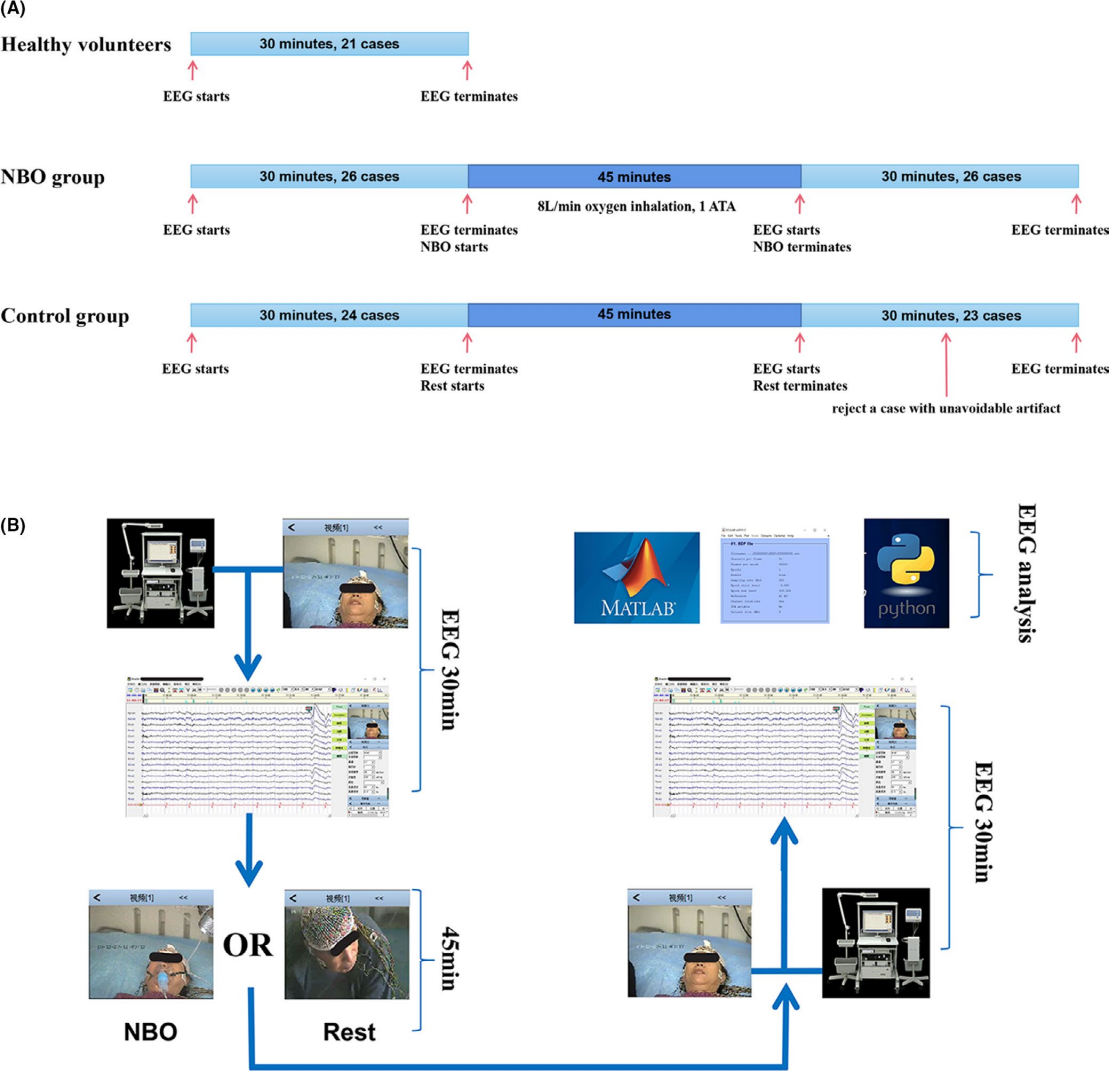
Study flow chart. (A) A total of 50 patients with CCI and 21 healthy volunteers underwent 30‐minute EEG recordings. (B) After that, the patients in NBO group (26 cases) underwent 8L/min oxygen inhalation, 1 ATA for 45 minutes, on the contrary, the patients in control group (24 cases) underwent a resting in room air for 45 minutes, like lying, sitting or walking without any specific interventions. And then, all of the CCI patients underwent the second time of 30‐minute EEG recordings again. One patient in control group with unavoidable second time of EEG artifact was rejected from final analysis; thus, there were 26 cases in NBO group and 23 cases in control group enrolling in the effective analysis finally

A subset of patients agreed to utilize NBO for an extended period (8 L/min, via simple mask, 45 min per time, and 3 times daily). After undergoing the long‐term NBO, the follow‐up EEG recordings were conducted as described above. A study flow chart is presented in Figure 1.

The EEG was recorded with the EEG‐recording equipment (EEG YAL PN‐NET, Beijing Yunshen Science and Technology Corporation). Ag/AgCl electrodes were positioned at Fp1, Fp2, F3, F4, F7, F8, Fz, C3, C4, Cz, P3, P4, Pz, T3, T4, T5, T6, O1, O2, A1, and A2 (21 channels) according to the international 10–20 system, and the electrode impedances were all less than 5 kΩ. The sampling rate was 256 Hz for all channels using a 16 bit AD convertor. Channel A1 and A2 were used as references. Five minutes of artifact‐free data were selected from EEG recordings in each subject at random, and these participants were maintained awake and lying quietly with eyes closed as much as possible. Data filtering (band pass, 1–30 Hz) was performed in EEGLAB (version 2019.1) with supplementary scripts operating in the MATLAB environment.^14^ The artifact removal (blink artifact and electromyogram artifact) was performed automatically using the BSS algorithm (sobi) that was implemented into EEGLAB software packages.

### 2.3 Outcomes

The absolute power (AP, with unit uV^2^) was computed using Fast Fourier Transform (FFT) for each electrode over the delta (1–4 Hz), theta (4–8 Hz), alpha (8–13 Hz), and beta (13–20 Hz) frequency bands, and the relative power (RP) was a ratio of the frequency band of interest power to the total power across the 1–20 Hz range. The power spectral density (PSD) for each channel was estimated via Welch's procedure with a 2‐s Hamming window length. The spectral power over the fronto‐central electrodes (F3, F4, Fz, C3, C4, and Cz), which were with less occipital alpha oscillation impact, was used to reflect the actual frequency activities in the forehead. The spectral power over the global electrodes (21 channels) was also presented as a reference. The fronto‐central alpha AP >1000 uV^2^ and ≤1000 uV^2^ were categorized as the high‐power EEG and the normal‐power EEG. Utilizing python software, the EEG wavelet entropy (with unit nat) was calculated via nonlinear dynamics method in order to evaluate the degree of brain injury.^15^ Power ratio index (PRI) was defined as AP ratios of theta/alpha (TAR), delta/alpha (DAR), (delta + theta)/(alpha + beta) (DTABR), and fronto‐central alpha/occipital alpha/3 (FOAR) in this study. For Part II, each frequency‐band AP and RP reduction rates were calculated as a ratio of the (pre‐ minus post‐intervention) band power to pre‐intervention band power.

### 2.4 Statistical analysis

R software (http://www.r‐project.org) was used for analysis in this study. Kolmogorov‐Smirnov test was used to assess the data distribution. Continuous data were expressed as mean ± standard deviation (SD) or median (interquartile range, IQR). Dataset following a Gaussian distribution was analyzed with Student's *t* test; otherwise analyzed with Mann‐Whitney U test. As for multiple comparisons among groups, LSD method was used to remove the statistical bias from repeated measure. The comparisons between the prior and post‐intervention parameters were performed with paired samples *t* test and Wilcoxon test. Categorical data were presented as numerical (percentage) and preceded by chi‐square test. Multiple analyses using a linear regression model were used to rule out the confounding effect of other covariates that may affect the EEG measures. *p*‐value < 0.05 was indicative of statistical significance.

## 3 RESULTS

### 3.1 Part I: the EEG anomalies in patients with CCI

A total of 50 eligible patients confirmed as CCI by perfusion imaging and 21 healthy volunteers were recruited in this study consecutively. Two senior doctors with extensive EEG experience finished the initial visual evaluations of the EEG maps and found that CCI patients had abnormal high voltage and amplitude waveforms over the forehead electrodes, and others had paroxysmal slow activities under the normal voltage background. Whereby, according to the fronto‐central alpha AP >1000 uV^2^ and ≤1000 uV^2^, the patients were considered to harbor high‐power EEG (26 cases) and the normal‐power EEG (24 cases). Baseline characteristics among the cohorts with the high‐power EEG, the normal‐power EEG, and the healthy EEG were similar, except for age and male percentage were lower in the healthy volunteers (Table 1).

**TABLE 1:** The clinical features of enrolled populations

	High‐power EEG	Normal‐power EEG	Health EEG
Demographics			
Num.	26	24	21
Age, yr	56.31±9.64	60.46±7.70	30.57±9.31
Male/female	21/5	16/8	4/17
Comorbid disease, n (%)			
Hypertension	16 (61.5)	15 (62.5)	NA
Diabetes	13 (50.0)	8 (33.3)	NA
Dyslipidemia	22 (84.6)	18 (75.0)	NA
Hyperuricemia	9 (34.6)	8 (33.3)	NA
Atrial fibrillation	2 (7.7)	1 (4.2)	NA
Coronary heart disease	8 (30.8)	3 (12.5)	NA
Clinical features			
Non‐focal neurological disorder, n (%)	21 (80.8)	22 (91.7)	NA
Focal neurological disorder, n (%)	3 (11.5)	5 (20.8)	NA
NIHSS	1 (0, 2)	1 (0.25, 2)	NA
mRS	0 (0, 1)	0 (0, 0)	NA
Imaging presentations, n (%)			
Unilateral anterior circulation stenosis	8 (30.8)	7 (29.2)	NA
Bilateral anterior circulation stenosis	16 (61.5)	16 (66.7)	NA
MCA stenosis	23 (88.5)	21 (87.5)	NA
ACA stenosis	4 (15.4)	6 (25.0)	NA
PCA stenosis	7 (26.9)	9 (37.5)	NA
VA stenosis	7 (26.9)	5 (20.8)	NA
BA stenosis	5 (19.2)	4 (16.7)	NA
ICA stenosis	10 (38.5)	9 (37.5)	NA
Brain infarction	1 (3.8)	1 (4.2)	NA
Scheltens scales, median (IQR)	4 (2, 6)	7 (3, 12.25)	NA

Abbreviation: NA, not available.

#### 3.1.1 Abnormal AP and RP

**FIGURE 2:**
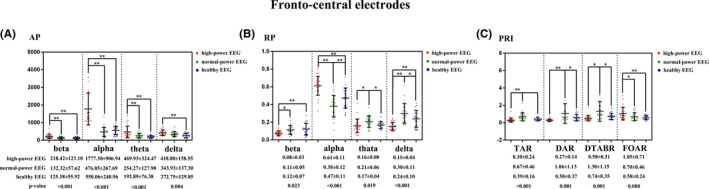
Differences in the spectral power and PRI among the high‐power EEG, normal‐power EEG, and healthy EEG. Mean±SD, *<0.05, **<0.01

Both the raw EEG and the PSD curves unraveled the remarkable differences among the high‐power EEG (n = 26), the normal‐power EEG (n = 24), and the healthy EEG (n = 21) (Figure S1). For the fronto‐central electrodes, the beta, alpha, theta, and delta AP in the high‐power EEG were substantially higher than those in the normal‐power EEG (*p* = 0.001, adjusted *p* < 0.001; *p* < 0.001, adjusted *p* < 0.001; *p* = 0.001, adjusted *p* = 0.004; *p* = 0.069, adjusted *p* = 0.044) and the healthy EEG (*p* = 0.001, adjusted *p* = 0.128; *p* < 0.001, adjusted *p* = 0.001; *p* < 0.001, adjusted *p* = 0.005; *p* = 0.001, adjusted *p* = 0.001). However, no difference was observed between the normal‐power EEG and the healthy EEG (all *p*>0.05). The alpha RP in the high‐power EEG was also higher than that in the normal‐power EEG (*p* < 0.001, adjusted *p* < 0.001) and the healthy EEG (*p* < 0.001, adjusted *p* = 0.053). In the normal‐power EEG, the theta and delta RP were significantly higher than those in the high‐power EEG (*p* = 0.010, adjusted *p* = 0.020; *p* < 0.001, adjusted *p* < 0.001) and the healthy EEG (*p* = 0.041, adjusted *p* = 0.253; *p* = 0.022, adjusted *p* = 0.064). However, the alpha RP was soundly lower than those in the high‐power EEG (*p*<0.001, adjusted *p*<0.001) and the healthy EEG (*p* = 0.009, adjusted *p* = 0.270). The differences in the global beta, alpha, theta, and delta AP and RP among the three types of EEG were similar to those over the fronto‐central electrodes aforementioned. Details were shown in Figure 2, and Tables S1‐S3.

#### 3.1.2 Abnormal PRI

The fronto‐central TAR, DAR, and DTABR in the normal‐power EEG were substantially higher than those in both the high‐power EEG (*p*<0.001, adjusted *p*<0.001; *p*<0.001, adjusted *p* = 0.001; *p*<0.001, adjusted *p* = 0.001) and the healthy EEG (*p* = 0.004, adjusted *p* = 0.043; *p* = 0.023, adjusted *p* = 0.034; *p* = 0.011, adjusted *p* = 0.016), but no difference could be found between the high‐power and the healthy EEG (all *p*>0.05, adjusted *p*>0.05). Meanwhile, the FOAR in the high‐power EEG was much higher than that in the normal‐power (*p* = 0.021, adjusted *p* = 0.065) and the healthy EEG (*p* = 0.003, adjusted *p* = 0.375). Furthermore, the global TAR, DAR, and DTABR in the normal‐power EEG were also greater than those in the high‐power EEG and the healthy EEG (all *p*<0.05, all adjusted *p*<0.05). Details were displayed in Figure 2 and Table S4.

#### 3.1.3 Abnormal wavelet entropy

The fronto‐central wavelet entropy in the CCI patients was statistically lower than that in the healthy volunteers (*p* = 0.055, adjusted *p* = 0.008). As for the CCI patients with high‐power EEG, the fronto‐central wavelet entropy was lower than that in both the CCI patients with normal‐power EEG (*p* = 0.001, adjusted *p* = 0.013) and the volunteers with healthy EEG (*p* = 0.001, adjusted *p* = 0.002), whereas the difference of the fronto‐central wavelet entropy between the CCI patients with normal‐power EEG and the volunteers with healthy EEG did not reach statistical significance (*p* = 0.859, adjusted *p* = 0.074). At the global areas of the brain, the statistical results were almost the same as the results mentioned above (see Table S5).

### 3.2 Part II: the effect of NBO on EEG anomalies

Forty‐nine out of 50 CCI patients finished the second time of 30‐minute EEG recording (one patient was rejected from final analysis due to the unavoidable artifact at the second time of EEG recording), in which 26 patients randomly entered into NBO group and all of them finished 45 minutes of NBO intervention during the interval of the two times of EEG recordings (baseline EEG recording showed that 15 cases of them presented as high‐power EEG and another 11 cases were normal‐power EEG); 23 patients randomly entered into control group underwent 45‐minute resting in room air during the interval of the two times of EEG recordings (baseline EEG recording revealed that 10 cases of them presented as high‐power EEG and other 13 cases were normal‐power EEG). These two arms were well‐matched in all baseline characteristics except for the ages. Details were illustrated in Table 2 and Table S6.

**TABLE 2:** Baseline characteristics of treatment groups

	NBO group	Control group	p‐value
Demographics			
Num.	26	23	NA
Age, yr	60.5±8.3	55.0±8.4	0.025
Male/female	18/8	19/4	0.277
Comorbid disease, n (%)			
Hypertension	14 (53.8)	16 (69.6)	0.260
Diabetes	11 (42.3)	10 (43.5)	0.934
Dyslipidemia	22 (84.6)	17 (73.9)	0.567
Hyperuricemia	10 (38.5)	7 (30.4)	0.556
Atrial fibrillation	2 (7.7)	1 (4.3)	1.000
Coronary heart disease	7 (26.9)	4 (17.4)	0.425
Clinical features			
Non‐focal neurological disorder, n (%)	22 (84.6)	20 (87.0)	1.000
Focal neurological disorder, n (%)	5 (19.2)	3 (13.0)	0.843
NIHSS	1 (0, 2)	1 (1, 2)	0.435
mRS	0 (0, 1)	0 (0, 0)	0.399
Imaging presentations, n (%)			
Unilateral anterior circulation stenosis	5 (19.2)	9 (39.1)	0.124
Bilateral anterior circulation stenosis	21 (80.8)	11 (47.8)	0.016
MCA stenosis	24 (92.3)	19 (82.6)	0.550
ACA stenosis	7 (26.9)	3 (13.0)	0.396
PCA stenosis	11 (42.3)	5 (21.7)	0.125
VA stenosis	7 (26.9)	5 (21.7)	0.674
BA stenosis	5 (19.2)	4 (17.4)	>0.999
ICA stenosis	12 (46.2)	7 (30.4)	0.260
Brain infarction	1 (3.8)	1 (4.3)	1.000
Scheltens scales, median (IQR)	5 (3, 9.5)	3 (2, 8)	0.347

Abbreviation: NA, not available.

#### 3.2.1 AP and RP correction

Among the CCI patients with high‐power EEG in NBO group (baseline vs. post‐NBO), NBO reduced the fronto‐central AP of the four frequency bands, but only the changes of the alpha AP reached significance (*p* = 0.042). However, the fronto‐central RP of the each frequency band did not change after NBO (all *p*>0.05). As for the patients with high‐power EEG in control group (baseline vs. post‐resting in room air), the fronto‐central beta AP (*p* = 0.01) and the theta RP (*p* = 0.018) increased significantly; both the AP and the RP of the other frequency bands were not changed (all *p*>0.05). In addition, the global AP of the four frequency bands tended to be reduced by NBO (all *p*>0.05) and the global theta RP was increased in control group (*p* = 0.001). An example of raw EEG and PSD was demonstrated in Figure S2.

**FIGURE 3:**
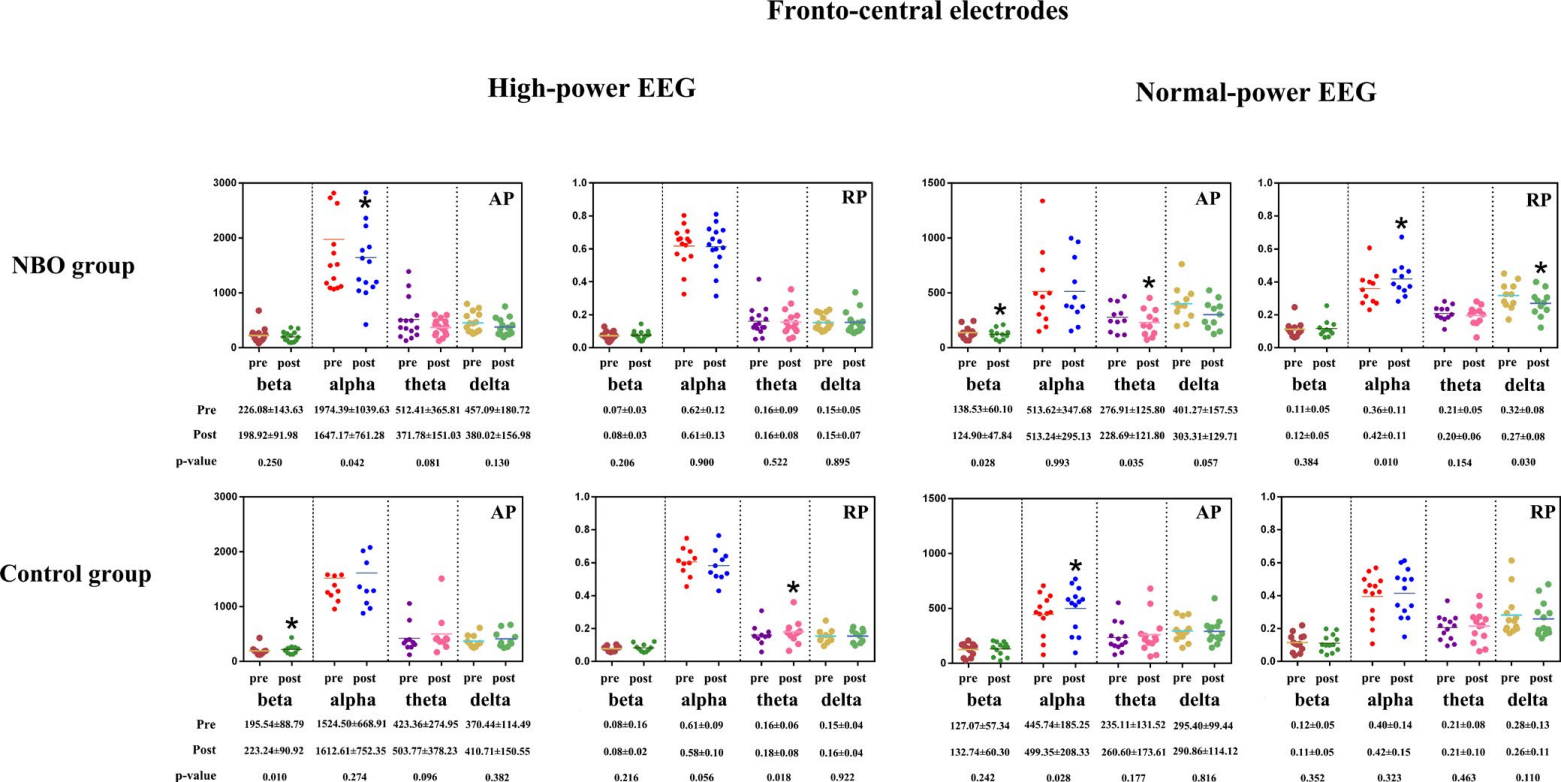
Spectral power changed in the NBO and control group. Median (IQR), *<0.05, **<0.01.

Among the CCI patients with normal‐power EEG in NBO group (Baseline vs. post‐NBO), the fronto‐central beta AP (*p* = 0.028), theta AP (*p* = 0.035), and delta RP (*p* = 0.030) declined, and alpha RP (*p* = 0.010) elevated after NBO. As for the patients with the normal‐power EEG in control group (Baseline vs. post‐resting in room air), only the fronto‐central alpha AP (*p* = 0.028) increased mildly. As for the global areas in the brain, the changes of neither the AP nor the RP of the frequency bands in both NBO and control groups showed no statistical significance (all *p*>0.05). Details were displayed in Figure 3 and Table S7.

#### 3.2.2 PRI correction

Among the CCI patients with high‐power EEG in NBO group (baseline vs. post‐NBO), the changes of all the fronto‐central TAR, DAR, and DTABR did not reach statistical significance, while the FOAR decreased obviously after NBO use (*p* = 0.041). As for control group (baseline vs. post‐resting in room air), only the TAR elevated after resting in room air (*p* = 0.017). Notably, the reduction rates of the fronto‐central beta AP, alpha AP, theta AP, and delta AP in NBO group were substantially higher than that in control group (*p* = 0.002, *p* = 0.047, *p* = 0.016, and *p* = 0.012, respectively). However, all of the reduction rates of the frequency‐band RP between NBO and control groups did not reach statistical significance (all *p*>0.05). Additionally, the global TAR, DAR, and DTABR did not change profoundly in NBO group; in contrast, the TAR in control group elevated substantially (*p* = 0.005). The reduction rates of the global theta AP and RP in NBO group were significantly higher than those in control group (*p* = 0.026; *p* = 0.012).

**FIGURE 4:**
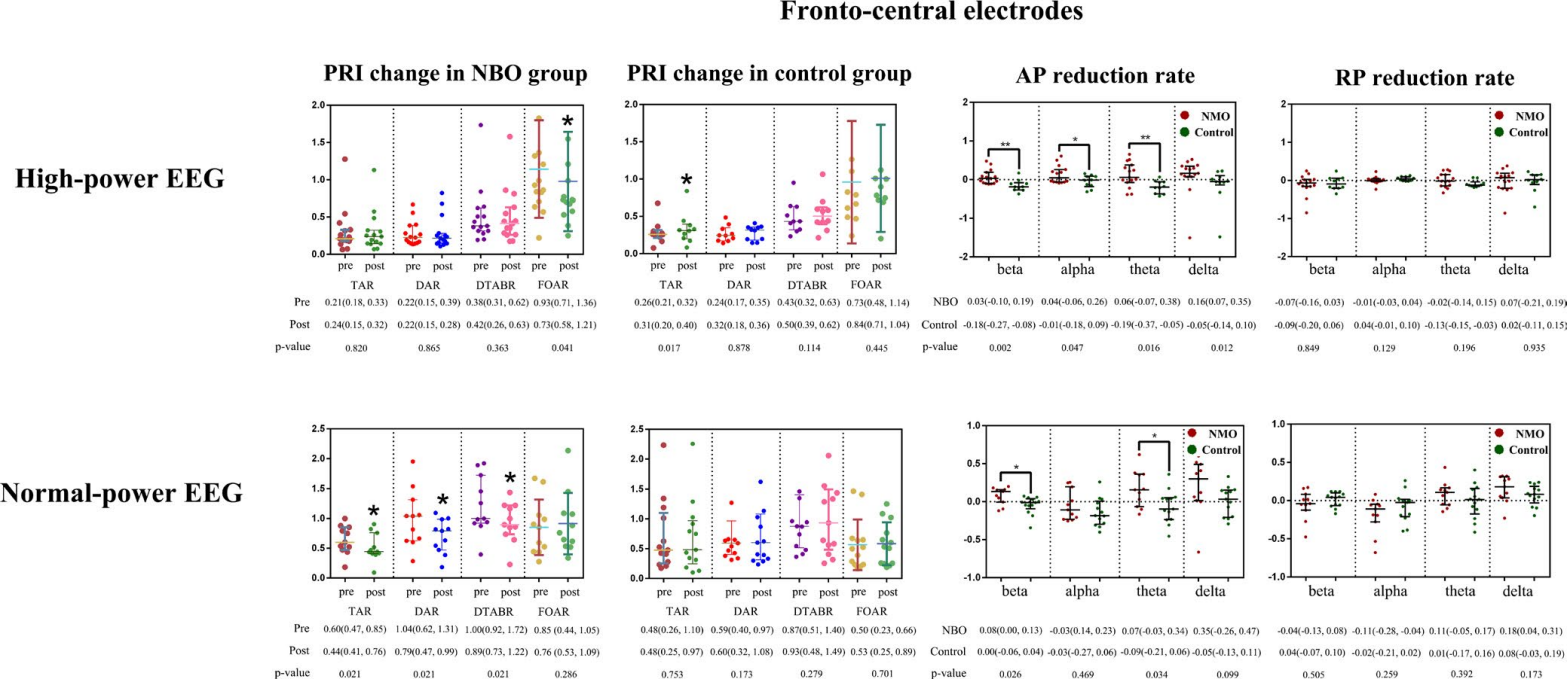
PRI changed and spectral power reduction rates in the NBO and control group. Median (IQR) and Mean±SD, *<0.05, **<0.01

For the CCI patients with normal‐power EEG (Baseline vs. post‐NBO), NBO significantly reduced the fronto‐central TAR (*p* = 0.021), DAR (*p* = 0.021), and DTABR (*p* = 0.021), whereas all these indexes showed no remarkable changes in control group (baseline vs. post‐resting in room air). The reduction rates of the beta AP (*p* = 0.012) and theta AP (*p* = 0.034) in NBO group were higher than those in control group. However, the reduction rates of RP between the two groups did not reach statistical significance (*p*>0.05). Moreover, the changes of the data of global TAR, DAR, DTABR, and FOAR in both NBO group and control did not reach statistical significance (all *p*>0.05). The reducing rates of global beta AP (*p* = 0.026) and theta AP (*p* = 0.034) in NBO group were greater than that in control group. Details can be found in Figure 4 and Table S8.

#### 3.2.3 Wavelet entropy change

As for both the high‐power EEG and the normal‐power EEG, the differences of the fronto‐central entropy (pre‐ minus post‐intervention) between NBO group and control group did not reach statistical significance; the global entropy between the two groups showed no statistical significance as well (all *p*>0.05). Details were shown in Table S9.

#### 3.2.4 The follow‐up EEG analysis

A total of seven patients who underwent NBO 45min/ 3 times daily for a median 4 months (range: 3–6 months) finished the follow‐up EEG recordings. Five out of the 7 patients were with the high‐power EEG, and 2 patients were with the normal‐power EEG at their baseline EEG maps. No patient complained of uncomfortable and adverse events onset during the period of continuous NBO intervention, each of them had a good compliance. As for the high‐power EEG, the fronto‐central alpha AP and RP were significantly reduced and the beta RP tended to be increased. All of above can be seen in Table S10.

## 4 DISCUSSION

Mounting evidence reveals NBO can increase the partial pressure of oxygen, attenuate neuron injury, improve oxidative metabolism, and reduce free radical damage. NBO application in cerebral ischemia is a topic of ongoing interest.^16^, ^17^, ^18^, ^19^, ^20^ The protective effect of NBO is mainly based on freezing ischemic penumbra.^12^, ^18^, ^21^ CCI is a prolonged state of hypoxic brain tissue, which may mimic the pathogenesis of ischemic penumbra in stroke.^1^, ^8^ Since NBO yields some benefit to the penumbra, in theory, it has the potential to play an ameliorative effect on CCI as well.^8^ As many CCI patients have non‐focal neurological symptoms (such as headache, dizziness, cognitive decline etc.), rather than focal neurological symptoms and signs, as we all know that the subjective neurological assessments (such as NIHSS, mRS, Barthel index, etc.) may be less useful for immediate real‐time NBO therapeutic assessment. On the contrary, EEG can reflect cerebral functional changes sensitively and dynamically.

EEG can reflect the extracellular currents in the dendrites of the cortical pyramidal cells; it is very sensitive for detecting cerebral ischemia and hypoxia.^22^, ^23^, ^24^, ^25^ Brain dysfunction can be represented on EEG through abnormal oscillation of certain frequencies.^26^, ^27^, ^28^, ^29^ Slow‐wave background (delta and theta activities) is reported as the most common findings in both acute and sub‐acute phases of stroke.^30^ However, few EEG features have been described for CCI up to now. This study delineated the abnormal EEG oscillations in patients with CCI for the first time and further investigated the efficacy of transient NBO on correcting CCI‐mediated EEG anomalies and also preliminary observed the long‐term NBO effects in some patients with CCI.

### 4.1 The anomalies of EEG in CCI patients

In this study, we found for the first time that the patients with CCI had obvious anomalies on EEG maps when compared with the healthy volunteers, which mainly presented as abnormal high‐power oscillations over the forehead electrodes and the paroxysmal slow activity release under the normal‐power background. According to this, the abnormal EEG of CCI was divided into the high‐power EEG and the normal‐power EEG.

Spectral power (AP and RP), which reflects the signal intensity, is generally computed for EEG activity of specific frequencies using FFT.^23^ The strong association of cerebral blood flow with spectral power has been well acknowledged.^25^, ^31^, ^32^ Slow background focal delta and theta activities were the most accepted EEG anomalies in cerebral ischemia.^23^ In this study, the quantitative results were in agreement with the visual evaluation for the raw EEG. The fronto‐central AP of each frequency in the high‐power EEG was profoundly higher than that in the healthy EEG and the normal‐power EEG. Abnormal high voltage and amplitude oscillations enhance the band power; thus, AP could represent the waveform directly. As for the normal‐power EEG, the theta and delta RP over the fronto‐central electrodes were prominently higher than that in both the healthy EEG and the high‐power EEG. The RP mainly reflects the components of the EEG oscillation, which means that more frequent slow activity release can cause higher theta and delta proportions over the whole power. However, the differences of slow frequency RP between the normal‐power EEG and the healthy EEG were diminished when adjusting for age and gender. The EEG oscillations in elderly people may slow down gradually,^33^, ^34^ whereby, whether CCI contributes to the higher RP of slow activities directly in the normal‐power EEG should be interpreted modestly.

Notably, the fronto‐central TAR, DAR, and DTABR, which represent the ratio of the slow activities to the fast activities, were the highest in the normal‐power EEG when compared with the healthy EEG and the high‐power EEG, even after adjusting for age and gender. This suggests that CCI could enhance the slow activity release under the normal‐power background, the EEG of which was completely different from the healthy EEG and the high‐power EEG regardless of age and gender discrepancy. Both the DAR and the DTABR are associated with clinical outcomes such as NIHSS and mRS scores, and they are also deemed as important indices in stroke patients.^35^, ^36^, ^37^ This study also corroborated that the DAR and DTABR could represent the clinically relevant features of the normal‐power EEG, even superior to theta and delta RP. We calculated the TAR, as a measurement index in this study, as we found that theta accounted for a significant proportion in slow activities under the normal‐power EEG. Due to the increment of power over all frequency bands, high‐power EEG did not present DAR, DTABR, and TAR elevation. The FOAR, which is the ratio of fronto‐central to occipital alpha power, was soundly higher in the high‐power EEG, compared with the normal‐power and the healthy EEG. Generally, alpha power at the forehead is substantially lower than that at the occipital lobe when people maintaining awake with eyes closed.^23^ By the comparison of the occipital alpha power, the FOAR could evaluate whether the forehead alpha power was extremely elevated. The results showed that the fronto‐central alpha power was definitely increased in the high‐power EEG, which corresponded with the AP analysis aforementioned.

The EEG signal complexity had been studied by means of wavelet entropy. Entropy analysis could provide a quantitative measure of the degree of brain injury and recovery.^15^ The entropy in the high‐power EEG was significantly lower than that in the normal‐power EEG and the healthy EEG, which indicated that brain injury of the patients with high‐power EEG would be more severe than that in the patients with normal‐power EEG.

### 4.2 The immediate effect of NBO on correcting EEG anomalies in CCI patients

In this study, we found that the EEG anomalies could be corrected by NBO immediately for the first time; that is, the high‐power oscillations were ameliorated and the paroxysmal slow activity release was controlled. Therefore, we concluded that NBO might improve the brain dysfunctions induced by CCI.

As discussed above, the frequency‐band AP and the FOAR may be more appropriate to reflect the high‐power condition; the frequency‐band RP, and TAR, DAR, and DTABR may represent the paroxysm slow activities under the normal‐power background. Therefore, these parameters were used to evaluate the effect of NBO on EEG anomalies. For the high‐power EEG in NBO group, the fronto‐central alpha AP and FOAR were significantly reduced, and the AP of the other frequency bands tended to be decreased. In control group, the theta RP and TAR were increased profoundly after resting, which might be caused by drowsiness. The fronto‐central AP reduction rates over the four frequency bands in NBO group were statistically higher than those in control group. These results strongly demonstrate that NBO application to the CCI patients with the high‐power EEG could ameliorate their abnormal high‐power oscillations immediately. As for the normal‐power EEG, NBO reduced the fronto‐central delta RP, and TAR, DAR, and DTABR significantly meanwhile elevated the alpha RP as well. However, all these parameters did not change in control group after resting. These results unraveled the paroxysmal slow activities release under the normal‐power background could be suppressed by NBO remarkably. The wavelet entropy did not change after NBO use. The EEG complexity is associated with brain injury, which cannot be alleviated by short‐term NBO.^15^

### 4.3 The long‐term effect of NBO on correcting EEG anomalies in CCI patients

After long‐term NBO use, the patients did not complain of any uncomfortable feelings, which indicated that long‐term NBO application to CCI patients was safe. Only five CCI patients with high‐power EEG were followed‐up, in whom the fronto‐central alpha AP and RP were significantly reduced. NBO in these patients could ameliorate the abnormal high‐power waveform over a long‐term intervention. Only two CCI patients with normal‐power EEG were available for follow‐up; thus, the long‐term EEG outcomes cannot provide a confirmatory result for this cohort.

### 4.4 Limitations

Firstly, the small sample size may bias the results toward null hypothesis. Especially for the patients with long‐term NBO use, the improvement of EEG anomalies may be more obvious after enlarging the sample size. Secondly, the age in healthy volunteers was not well‐matched with that in the CCI patients. Healthy controls are too difficult to be found in aged populations due to the high incidence of CCI.^1^ A larger recruitment may offset this mismatch in the future. However, in general, the voltage and power of oscillations declined gradually with age^33^, ^34^; thus, the bias from age mismatch in this study tended to negate the actual differences between the CCI and the healthy control. Meanwhile, we also performed multi‐variate analysis to rule out the confounding impact of age, in order to obtain the more accurate results. Thirdly, despite trying to select EEG epoch with patients maintaining awake and eyes closed, the drowsiness‐induced theta activity was inevitable under long periods of sitting/ lying, especially when patients rested in room air.

## 5 CONCLUSION

NBO can rapidly ameliorate CCI‐related EEG anomalies, including attenuating the abnormal high‐power oscillations and suppressing the paroxysmal slow activities in the CCI condition. Long‐term NBO performance may still benefit in the CCI patients to some extent. NBO may be a promising approach to protect the brain from CCI‐induced cerebral dysfunctions.

## CONFLICT OF INTEREST

JD, YL, GR, ZC, SZ, YD, XJ, and RM report no conflicts of interest.

## AUTHORS’ CONTRIBUTIONS

J.‐Y.D., Y.L., and R.M. formulated the conception and design of the study, drafted the manuscript, and prepared the figures; Z.‐Y.C., S.‐Y.Z., J.‐Y.D., and R.M. contributed to data acquisition; Y.L. and S.‐Y.Z. recorded the EEG maps; J.‐Y.D. and Z.‐Y.C. were responsible for the EEG data analysis; G.‐B.R. and Y.‐C.D. made critical revisions of the manuscript; G.‐B.R., Y.‐C.D., and X.‐M.J. gave the pivotal advices for the study design, data interpretation, and statistical analysis. G.‐B.R and Y.‐C.D. refined the use of English.

## Supporting information

Supplementary Material

## Data Availability

The data presented in the study are available in the supplementary material of this article. Further inquiries can be directed to the corresponding authors.

## References

[1] Zhou DA, Meng R, Li SJ, et al. Advances in chronic cerebral circulation insufficiency. CNS Neurosci Ther. 2018;24:5‐17. 29143463 10.1111/cns.12780 PMC6489997

[2] Wang Y, Zhao X, Liu L, et al. Prevalence and outcomes of symptomatic intracranial large artery stenoses and occlusions in China: the Chinese Intracranial Atherosclerosis (CICAS) Study. Stroke. 2014;45:663‐669. 24481975 10.1161/STROKEAHA.113.003508

[3] Wong LK. Global burden of intracranial atherosclerosis. Int J Stroke. 2006;1:158‐159. 18706036 10.1111/j.1747-4949.2006.00045.x

[4] Chu DK, Kim LH, Young PJ, et al. Mortality and morbidity in acutely ill adults treated with liberal versus conservative oxygen therapy (IOTA): a systematic review and meta‐analysis. Lancet. 2018;391:1693‐1705. 29726345 10.1016/S0140-6736(18)30479-3

[5] Ding J, Zhou DA, Sui M, et al. The effect of normobaric oxygen in patients with acute stroke: a systematic review and meta‐analysis. Neurol Res. 2018;40:433‐444. 29600891 10.1080/01616412.2018.1454091

[6] Singhal AB, Benner T, Roccatagliata L, et al. A pilot study of normobaric oxygen therapy in acute ischemic stroke. Stroke. 2005;36:797‐802. 15761201 10.1161/01.STR.0000158914.66827.2e

[7] Roffe C, Ali K, Warusevitane A, et al. The SOS pilot study: a RCT of routine oxygen supplementation early after acute stroke–effect on recovery of neurological function at one week. PLoS One. 2011;6:e19113. 21625533 10.1371/journal.pone.0019113 PMC3098237

[8] Ding J, Zhou D, Liu C, et al. Normobaric oxygen: a novel approach for treating chronic cerebral circulation insufficiency. Clin Interv Aging. 2019;14:565‐570. 30936686 10.2147/CIA.S190984 PMC6421875

[9] Singhal AB, Ratai E, Benner T, et al. Magnetic resonance spectroscopy study of oxygen therapy in ischemic stroke. Stroke. 2007;38:2851‐2854. 17761914 10.1161/STROKEAHA.107.487280

[10] Rønning OM, Guldvog B. Should stroke victims routinely receive supplemental oxygen? A quasi‐randomized controlled trial. Stroke. 1999;30:2033‐2037. 10512903 10.1161/01.str.30.10.2033

[11] Shin HK, Dunn AK, Jones PB, et al. Normobaric hyperoxia improves cerebral blood flow and oxygenation, and inhibits peri‐infarct depolarizations in experimental focal ischaemia. Brain. 2007;130:1631‐1642. 17468117 10.1093/brain/awm071 PMC3023418

[12] Liang J, Qi Z, Liu W, et al. Normobaric hyperoxia slows blood‐brain barrier damage and expands the therapeutic time window for tissue‐type plasminogen activator treatment in cerebral ischemia. Stroke. 2015;46:1344‐1351. 25804925 10.1161/STROKEAHA.114.008599 PMC4414814

[13] Shi S, Qi Z, Ma Q, et al. Normobaric hyperoxia reduces blood occludin fragments in rats and patients with acute ischemic stroke. Stroke. 2017;48:2848‐2854. 28931617 10.1161/STROKEAHA.117.017713 PMC5659343

[14] Delorme A, Makeig S. EEGLAB: an open source toolbox for analysis of single‐trial EEG dynamics including independent component analysis. J Neurosci Methods. 2004;134:9‐21. 15102499 10.1016/j.jneumeth.2003.10.009

[15] Bezerianos A, Tong S, Thakor N. Time‐dependent entropy estimation of EEG rhythm changes following brain ischemia. Ann Biomed Eng. 2003;31:221‐232. 12627829 10.1114/1.1541013

[16] Ejaz S, Emmrich JV, Sitnikov SL, et al. Normobaric hyperoxia markedly reduces brain damage and sensorimotor deficits following brief focal ischaemia. Brain. 2016;139:751‐764. 26767570 10.1093/brain/awv391

[17] Ivanov KP, Sokolova IB, Vovenko EP. Oxygen transport in the rat brain cortex at normobaric hyperoxia. Eur J Appl Physiol Occup Physiol. 1999;80:582‐587. 10541925 10.1007/s004210050637

[18] Liu S, Shi H, Liu W, Furuichi T, Timmins GS, Liu KJ. Interstitial pO2 in ischemic penumbra and core are differentially affected following transient focal cerebral ischemia in rats. J Cereb Blood Flow Metab. 2004;24:343‐349. 15091115 10.1097/01.WCB.0000110047.43905.01

[19] Poli S, Veltkamp R. Oxygen therapy in acute ischemic stroke ‐ experimental efficacy and molecular mechanisms. Curr Mol Med. 2009;9:227‐241. 19275631 10.2174/156652409787581619

[20] Sun L, Wolferts G, Veltkamp R. Oxygen therapy does not increase production and damage induced by reactive oxygen species in focal cerebral ischemia. Neurosci Lett. 2014;577:1‐5. 24909618 10.1016/j.neulet.2014.05.060

[21] Xu JI, Zhang Y, Liang Z, et al. Normobaric hyperoxia retards the evolution of ischemic brain tissue toward infarction in a rat model of transient focal cerebral ischemia. Neurol Res. 2016;38:75‐79. 27078693 10.1080/01616412.2015.1135558

[22] Evans BM. Patterns of arousal in comatose patients. J Neurol Neurosurg Psychiatry. 1976;39:392‐402. 932756 10.1136/jnnp.39.4.392 PMC492291

[23] Finnigan S, van Putten MJ. EEG in ischaemic stroke: quantitative EEG can uniquely inform (sub‐)acute prognoses and clinical management. Clin Neurophysiol. 2013;124:10‐19. 22858178 10.1016/j.clinph.2012.07.003

[24] Foreman B, Claassen J. Quantitative EEG for the detection of brain ischemia. Crit Care. 2012;16:216. 22429809 10.1186/cc11230 PMC3681361

[25] Jordan KG. Emergency EEG and continuous EEG monitoring in acute ischemic stroke. J Clin Neurophysiol. 2004;21:341‐352. 15592008

[26] Assenza G, Zappasodi F, Squitti R, et al. Neuronal functionality assessed by magnetoencephalography is related to oxidative stress system in acute ischemic stroke. NeuroImage. 2009;44:1267‐1273. 19010427 10.1016/j.neuroimage.2008.09.049

[27] Hsiao FJ, Wang YJ, Yan SH, Chen WT, Lin YY. Altered oscillation and synchronization of default‐mode network activity in mild Alzheimer's disease compared to mild cognitive impairment: an electrophysiological study. PLoS One. 2013;8:e68792. 23874766 10.1371/journal.pone.0068792 PMC3708894

[28] Moretti DV, Prestia A, Binetti G, Zanetti O, Frisoni GB. Increase of theta frequency is associated with reduction in regional cerebral blood flow only in subjects with mild cognitive impairment with higher upper alpha/low alpha EEG frequency power ratio. Front Behav Neurosci. 2013;7:188. 24367305 10.3389/fnbeh.2013.00188 PMC3851738

[29] Roh JH, Park MH, Ko D, et al. Region and frequency specific changes of spectral power in Alzheimer's disease and mild cognitive impairment. Clin Neurophysiol. 2011;122:2169‐2176. 21715226 10.1016/j.clinph.2011.03.023

[30] Finnigan SP, Rose SE, Walsh M, et al. Correlation of quantitative EEG in acute ischemic stroke with 30‐day NIHSS score: comparison with diffusion and perfusion MRI. Stroke. 2004;35:899‐903. 15001786 10.1161/01.STR.0000122622.73916.d2

[31] Sundt TM Jr, Sharbrough FW, Anderson RE, Michenfelder JD. Cerebral blood flow measurements and electroencephalograms during carotid endarterectomy. J Neurosurg. 1974;41:310‐320. 4412366 10.3171/jns.1974.41.3.0310

[32] Sundt TM Jr, Sharbrough FW, Piepgras DG, Kearns TP, Messick JM Jr, O'Fallon WM. Correlation of cerebral blood flow and electroencephalographic changes during carotid endarterectomy: with results of surgery and hemodynamics of cerebral ischemia. Mayo Clin Proc. 1981;56:533‐543. 7266064

[33] Dustman RE, Shearer DE, Emmerson RY. EEG and event‐related potentials in normal aging. Prog Neurogibol. 1993;41:369‐401. 10.1016/0301-0082(93)90005-d 8210412

[34] Tatum WO 4th, Husain AM, Benbadis SR, Kaplan PW. Normal adult EEG and patterns of uncertain significance. J Clin Neurophysiol. 2006;23:194‐207. 16751720 10.1097/01.wnp.0000220110.92126.a6

[35] Finnigan SP, Walsh M, Rose SE, Chalk JB. Quantitative EEG indices of sub‐acute ischaemic stroke correlate with clinical outcomes. Clin Neurophysiol. 2007;118:2525‐2532. 17889600 10.1016/j.clinph.2007.07.021

[36] Leon‐Carrion J, Martin‐Rodriguez JF, Damas‐Lopez J, Barroso y Martin JM, Dominguez‐Morales MR. Delta‐alpha ratio correlates with level of recovery after neurorehabilitation in patients with acquired brain injury. Clin Neurophysiol. 2009;120:1039‐1045. 19398371 10.1016/j.clinph.2009.01.021

[37] Sheorajpanday RV, Nagels G, Weeren AJ, De Surgeloose D, De Deyn PP. Additional value of quantitative EEG in acute anterior circulation syndrome of presumed ischemic origin. Clin Neurophysiol. 2010;121:1719‐1725. 20181521 10.1016/j.clinph.2009.10.037

