# Supplementary material for: Normobaric oxygen may correct chronic cerebral ischemia‐mediated EEG anomalies
Source: CNS Neurosci Ther. 2021 Jul 9;27(10):1214–23. doi: 10.1111/cns.13703 (PMC8446210; doi:10.1111/cns.13703)
Supplement: Supplementary file 1 — Supplementary Material [file CNS-27-1214-s001.doc]

**SUPPLEMENTAL MATERIAL**

**Normobaric oxygen may correct chronic cerebral ischemia induced EEG anomalies**

Jiayue Ding, MD^1, 2^*, Yu Liu, MD^3^*, Gary B. Rajah, MD^4^, Zhiying Chen, MD^1, 5, 6^, Shiyong Zhang, MD, PhD^7^, Yuchuan Ding, MD, PhD^6,8^, Xunming Ji, MD, PhD^5, 6, 8^, Ran Meng, MD, PhD^1, 5, 6^

1. Department of Neurology, Xuanwu Hospital, Capital Medical University, Beijing, 100053, China
2. Department of Neurology, Tianjin Medical University General Hospital, Tianjin, 300052, China
3. Epilepsy Center, Beijing Fengtai You’anmen Hospital, Beijing, 100069, China
4. Department of Neurosurgery, Jacobs School of Medicine and Biomedical Sciences, University at Buffalo, Buffalo, New York, USA
5. Advanced Center of Stroke, Beijing Institute for Brain Disorders, Beijing, 100053, China
6. Department of China-America Institute of Neuroscience, Xuanwu Hospital, Capital Medical University, Beijing, 100053, China
7. Department of Interventional Neurology, Beijing Fengtai You’anmen Hospital, Beijing, 100069, China
8. Department of Neurosurgery, Wayne State University School of Medicine, Detroit, Michigan, 48201, USA

*These authors contributed equally to the manuscript.

**Supplementary tables**

**Table 1.** Healthy versus high-power EEG and the healthy EEG

|  | High-power EEG | Healthy EEG | LSD p-value | Adjusted p-value |
| --- | --- | --- | --- | --- |
| Global area |  |  |  |  |
| Beta AP | 668.55±345.50 | 477.87±218.70 | 0.016 | 0.385 |
| Beta RP | 0.08±0.02 | 0.14±0.10 | <0.001 | 0.002 |
| Alpha AP | 5496.39±2770.00 | 2131.80±1374.09 | <0.001 | 0.005 |
| Alpha RP | 0.62±0.10 | 0.51±0.12 | 0.004 | 0.076 |
| Theta AP | 1247.03±781.73 | 528.60±222.71 | <0.001 | 0.002 |
| Theta RP | 0.14±0.07 | 0.14±0.04 | 0.700 | 0.276 |
| Delta AP | 1343.50±475.11 | 799.74±405.39 | <0.001 | 0.002 |
| Delta RP | 0.16±0.04 | 0.21±0.09 | 0.077 | 0.515 |
| Fronto-central area |  |  |  |  |
| Beta AP | 218.42±123.10 | 125.38±55.92 | 0.001 | 0.128 |
| Beta RP | 0.08±0.03 | 0.12±0.07 | 0.003 | 0.001 |
| Alpha AP | 1777.30±906.94 | 550.06±240.96 | <0.001 | 0.001 |
| Alpha RP | 0.61±0.11 | 0.47±0.11 | <0.001 | 0.053 |
| Theta AP | 469.93±324.47 | 192.89±76.38 | <0.001 | 0.005 |
| Theta RP | 0.16±0.08 | 0.17±0.04 | 0.661 | 0.587 |
| Delta AP | 418.88±158.55 | 272.75±129.85 | 0.001 | 0.001 |
| Delta RP | 0.15±0.04 | 0.24±0.10 | 0.001 | 0.204 |
| Adjusted p-value is adjusted for age and gender. | | | | |

**Table 2.** The differences of each frequency band power between the normal-power EEG and the healthy EEG

|  | Normal-power EEG | Healthy EEG | LSD p-value | Adjusted p-value |
| --- | --- | --- | --- | --- |
| Global area |  |  |  |  |
| Beta AP | 406.45±181.86 | 477.87±218.70 | 0.367 | 0.046 |
| Beta RP | 0.10±0.05 | 0.14±0.10 | 0.030 | 0.039 |
| Alpha AP | 1881.73±1063.19 | 2131.80±1374.09 | 0.667 | 0.736 |
| Alpha RP | 0.44±0.15 | 0.51±0.12 | 0.058 | 0.288 |
| Theta AP | 687.49±336.74 | 528.60±222.71 | 0.316 | 0.583 |
| Theta RP | 0.17±0.06 | 0.14±0.04 | 0.045 | 0.093 |
| Delta AP | 1052.04±395.46 | 799.74±405.39 | 0.053 | 0.356 |
| Delta RP | 0.28±0.12 | 0.21±0.09 | 0.006 | 0.046 |
| Fronto-central area |  |  |  |  |
| Beta AP | 132.32±57.62 | 125.38±55.92 | 0.791 | 0.083 |
| Beta RP | 0.11±0.05 | 0.12±0.07 | 0.585 | 0.031 |
| Alpha AP | 476.85±267.69 | 550.06±240.96 | 0.677 | 0.857 |
| Alpha RP | 0.38±0.12 | 0.47±0.11 | 0.009 | 0.270 |
| Theta AP | 254.27±127.90 | 192.89±76.38 | 0.341 | 0.559 |
| Theta RP | 0.21±0.06 | 0.17±0.04 | 0.041 | 0.253 |
| Delta AP | 343.93±137.30 | 272.75±129.85 | 0.101 | 0.299 |
| Delta RP | 0.30±0.11 | 0.24±0.10 | 0.022 | 0.064 |
| Adjusted p-value is adjusted for age and gender. | | | | |

**Table 3.** The differences of the each frequency band power between the high-power EEG and normal-power EEG

|  | High-power EEG | Normal-power EEG | LSD p-value | Adjusted p-value |
| --- | --- | --- | --- | --- |
| Global area |  |  |  |  |
| Beta AP | 668.55±345.50 | 406.45±181.86 | 0.001 | <0.001 |
| Beta RP | 0.08±0.02 | 0.10±0.05 | 0.138 | 0.052 |
| Alpha AP | 5496.39±2770.00 | 1881.73±1063.19 | <0.001 | <0.001 |
| Alpha RP | 0.62±0.10 | 0.44±0.15 | <0.001 | <0.001 |
| Theta AP | 1247.03±781.73 | 687.49±336.74 | <0.001 | 0.002 |
| Theta RP | 0.14±0.07 | 0.17±0.06 | 0.083 | 0.125 |
| Delta AP | 1343.50±475.11 | 1052.04±395.46 | 0.019 | 0.014 |
| Delta RP | 0.16±0.04 | 0.28±0.12 | <0.001 | <0.001 |
| Fronto-central area |  |  |  |  |
| Beta AP | 218.42±123.10 | 132.32±57.62 | 0.001 | <0.001 |
| Beta RP | 0.08±0.03 | 0.11±0.05 | 0.010 | 0.012 |
| Alpha AP | 1777.30±906.94 | 476.85±267.69 | <0.001 | <0.001 |
| Alpha RP | 0.61±0.11 | 0.38±0.12 | <0.001 | <0.001 |
| Theta AP | 469.93±324.47 | 254.27±127.90 | 0.001 | 0.004 |
| Theta RP | 0.16±0.08 | 0.21±0.06 | 0.010 | 0.020 |
| Delta AP | 418.88±158.55 | 343.93±137.30 | 0.069 | 0.044 |
| Delta RP | 0.15±0.04 | 0.30±0.11 | <0.001 | <0.001 |
| Adjusted p-value is adjusted for age and gender. | | | | |

**Table 4.** The PRI between the high-power EEG and the normal-power EEG

|  | High-power EEG | Healthy EEG | LSD p-value | Adjusted p-value |
| --- | --- | --- | --- | --- |
| Global area |  |  |  |  |
| TAR | 0.27±0.23 | 0.29±0.12 | 0.750 | 0.891 |
| DAR | 0.28±0.14 | 0.46±0.31 | 0.299 | 0.299 |
| DTABR | 0.48±0.30 | 0.58±0.30 | 0.599 | 0.869 |
| FOAR | 1.05±0.71 | 0.58±0.24 | 0.003 | 0.375 |
| Fronto-central area |  |  |  |  |
| TAR | 0.30±0.24 | 0.39±0.16 | 0.324 | 0.434 |
| DAR | 0.27±0.14 | 0.58±0.37 | 0.135 | 0.062 |
| DTABR | 0.50±0.31 | 0.74±0.35 | 0.249 | 0.572 |
|  | Normal-power EEG | Healthy EEG |  |  |
| Global area |  |  |  |  |
| TAR | 0.51±0.40 | 0.29±0.12 | 0.010 | 0.023 |
| DAR | 0.90±0.95 | 0.46±0.31 | 0.015 | 0.025 |
| DTABR | 1.09±0.97 | 0.58±0.30 | 0.007 | 0.013 |
| FOAR | 0.70±0.46 | 0.58±0.24 | 0.445 | 0.284 |
| Fronto-central area |  |  |  |  |
| TAR | 0.67±0.46 | 0.39±0.16 | 0.004 | 0.043 |
| DAR | 1.06±1.13 | 0.58±0.37 | 0.023 | 0.034 |
| DTABR | 1.30±1.15 | 0.74±0.35 | 0.011 | 0.016 |
|  | Normal-power EEG | High-power EEG |  |  |
| Global area |  |  |  |  |
| TAR | 0.51±0.40 | 0.27±0.23 | 0.003 | 0.008 |
| DAR | 0.90±0.95 | 0.28±0.14 | <0.001 | 0.001 |
| DTABR | 1.09±0.97 | 0.48±0.30 | 0.001 | 0.002 |
| FOAR | 0.70±0.46 | 1.05±0.71 | 0.021 | 0.065 |
| Fronto-central area |  |  |  |  |
| TAR | 0.67±0.46 | 0.30±0.24 | <0.001 | <0.001 |
| DAR | 1.06±1.13 | 0.27±0.14 | <0.001 | 0.001 |
| DTABR | 1.30±1.15 | 0.50±0.31 | <0.001 | 0.001 |
| Adjusted p-value is adjusted for age and gender. | | | | |

**Table 5.** The wavelet entropy among the three types of EEG

|  | High-power EEG | Healthy EEG | LSD p-value | Adjusted p-value |
| --- | --- | --- | --- | --- |
| Global area | 34.28±2.19 | 36.46±1.95 | 0.001 | 0.003 |
| Fronto-central area | 9.78±0.71 | 10.47±0.58 | 0.001 | 0.002 |
|  | Normal-power EEG | Healthy EEG |  |  |
| Global area | 35.98±2.40 | 36.46±1.95 | 0.472 | 0.178 |
| Fronto-central area | 10.44±0.77 | 10.47±0.58 | 0.859 | 0.074 |
|  | High-power EEG | Normal-power EEG |  |  |
| Global area | 34.28±2.19 | 35.98±2.40 | 0.008 | 0.035 |
| Fronto-central area | 9.78±0.71 | 10.44±0.77 | 0.001 | 0.013 |
| Adjusted p-value is adjusted for age and gender. | | | | |

**Table 6.** Baseline characteristics of treatment groups

|  | NBO group | | Control group | |
| --- | --- | --- | --- | --- |
|  | High-power EEG | Normal-power EEG | High-power EEG | Normal-power EEG |
| Demographics |  |  |  |  |
| Num. | 15 | 11 | 10 | 13 |
| Age, yr | 60.20±9.91 | 67.36±10.00 | 52.80±9.13 | 58.54±7.07 |
| Male/female | 11/4 | 7/4 | 10/0 | 9/4 |
| Comorbid disease, n (%) |  |  |  |  |
| Hypertension | 9 (60.0) | 5 (45.5) | 6 (60.0) | 10 (76.9) |
| Diabetes | 7 (46.7) | 4 (36.4) | 6 (60.0) | 4 (30.8) |
| Dyslipidemia | 13 (86.7) | 9 (81.8) | 8 (80.0) | 9 (69.2) |
| Hyperuricemia | 5 (33.3) | 5 (45.5) | 4 (40.0) | 3 (23.1) |
| Atrial fibrillation | 1 (6.7) | 1 (9.1) | 1 (10.0) | 0 (0.0) |
| Coronary heart disease | 5 (33.3) | 2 (18.2) | 3 (30.0) | 1 (7.7) |
| Clinical features |  |  |  |  |
| Non-focal neurological disorder, n (%) | 12 (80.0) | 10 (90.9) | 8 (80.0) | 12 (92.3) |
| Focal neurological disorder, n (%) | 2 (13.3) | 3 (27.3) | 1 (10.0) | 2 (15.4) |
| NIHSS | 1 (0, 2) | 1 (0, 2) | 1 (0, 2) | 2 (1, 2) |
| mRS | 0 (0, 1) | 0 (0, 0) | 0 (0, 0) | 0 (0, 0.5) |
| Imaging presentations, n (%) |  |  |  |  |
| Unilateral anterior circulation stenosis | 4 (26.7) | 1 (9.1) | 3 (30.0) | 6 (46.2) |
| Bilateral anterior circulation stenosis | 11 (73.3) | 10 (90.9) | 5 (50.0) | 6 (46.2) |
| MCA stenosis | 15 (100.0) | 9 (81.8) | 7 (70.0) | 12 (92.3) |
| ACA stenosis | 4 (26.7) | 3 (27.3) | 0 (0.0) | 3 (23.1) |
| PCA stenosis | 6 (40.0) | 5 (45.5) | 1 (10.0) | 4 (30.8) |
| VA stenosis | 4 (26.7) | 3 (27.3) | 3 (30.0) | 2 (15.4) |
| BA stenosis | 3 (20.0) | 2 (18.2) | 2 (20.0) | 2 (15.4) |
| ICA stenosis | 7 (46.7) | 5 (45.5) | 3 (30.0) | 4 (30.8) |
| Brain infarction | 1 (6.7) | 0 (0.0) | 0 (0.0) | 1 (7.7) |
| Scheltens scales, median (IQR) | 4 (2, 6) | 8.5 (3, 14) | 2 (1, 6) | 4 (2, 10.75) |
| NA: no available. | | | |  |

**Table 7.** The power changes in the NBO and control group

| High-power EEG | Global area | | | Fronto-central area | | |
| --- | --- | --- | --- | --- | --- | --- |
| Pre-intervention | NBO group | Control group | p-value | NBO group | Control group | p-value |
| Beta AP | 682.67±405.91 | 623.71±257.86 | 0.688 | 226.08±143.63 | 195.54±88.79 | 0.556 |
| Beta RP | 0.07±0.03 | 0.08±0.01 | 0.538 | 0.07±0.03 | 0.08±0.16 | 0.502 |
| Alpha AP | 5842.85±3133.87 | 5052.57±2356.56 | 0.505 | 1974.39±1039.63 | 1524.50±668.91 | 0.240 |
| Alpha RP | 0.61±0.12 | 0.62±0.08 | 0.769 | 0.62±0.12 | 0.61±0.09 | 0.819 |
| Theta AP | 1376.66±875.84 | 1094.81±656.29 | 0.395 | 512.41±365.81 | 423.36±274.95 | 0.519 |
| Theta RP | 0.15±0.08 | 0.14±0.06 | 0.665 | 0.16±0.09 | 0.16±0.06 | 0.949 |
| Delta AP | 1459.84±551.17 | 1201.36±312.74 | 0.193 | 457.09±180.72 | 370.44±114.49 | 0.193 |
| Delta RP | 0.17±0.05 | 0.16±0.04 | 0.790 | 0.15±0.05 | 0.15±0.04 | 0.918 |
| Post-intervention |  |  |  |  |  |  |
| Beta AP | 621.82±324.43 | 672.73±253.15 | 0.680 | 198.92±91.98 | 223.24±90.92 | 0.522 |
| Beta RP | 0.08±0.02 | 0.09±0.02 | 0.304 | 0.08±0.03 | 0.08±0.02 | 0.506 |
| Alpha AP | 5279.60±2554.50 | 5126.14±2471.58 | 0.883 | 1647.17±761.28 | 1612.61±752.35 | 0.912 |
| Alpha RP | 0.62±0.13 | 0.61±0.08 | 0.821 | 0.61±0.13 | 0.58±0.10 | 0.512 |
| Theta AP | 1030.08±410.13 | 1235.74±840.82 | 0.422 | 371.78±151.03 | 503.77±378.23 | 0.234 |
| Theta RP | 0.14±0.07 | 0.15±0.06 | 0.675 | 0.16±0.08 | 0.18±0.08 | 0.467 |
| Delta AP | 1234.23±434.82 | 1159.70±329.67 | 0.650 | 380.02±156.98 | 410.71±150.55 | 0.631 |
| Delta RP | 0.16±0.06 | 0.15±0.03 | 0.567 | 0.15±0.07 | 0.16±0.04 | 0.960 |
| Normal-power EEG |  |  |  |  |  |  |
| Pre-intervention |  |  |  |  |  |  |
| Beta AP | 403.65±165.04 | 408.82±201.69 | 0.946 | 138.53±60.10 | 127.07±57.34 | 0.638 |
| Beta RP | 0.11±0.06 | 0.10±0.04 | 0.789 | 0.11±0.05 | 0.12±0.05 | 0.891 |
| Alpha AP | 1768.48±1114.60 | 1977.55±1053.36 | 0.642 | 513.62±347.68 | 445.74±185.25 | 0.548 |
| Alpha RP | 0.40±0.11 | 0.47±0.17 | 0.221 | 0.36±0.11 | 0.40±0.14 | 0.487 |
| Theta AP | 743.82±342.09 | 639.82±338.29 | 0.463 | 276.91±125.80 | 235.11±131.52 | 0.437 |
| Theta RP | 0.18±0.04 | 0.17±0.07 | 0.690 | 0.21±0.05 | 0.21±0.08 | 0.887 |
| Delta AP | 1252.11±463.29 | 882.75±230.52 | 0.019 | 401.27±157.53 | 295.40±99.44 | 0.058 |
| Delta RP | 0.32±0.08 | 0.26±0.14 | 0.218 | 0.32±0.08 | 0.28±0.13 | 0.448 |
| Post-intervention |  |  |  |  |  |  |
| Beta AP | 368.03±136.73 | 420.51±205.51 | 0.478 | 124.90±47.84 | 132.74±60.30 | 0.731 |
| Beta RP | 0.11±0.06 | 0.10±0.04 | 0.536 | 0.12±0.05 | 0.11±0.05 | 0.819 |
| Alpha AP | 1703.80±1012.63 | 2116.30±1228.59 | 0.385 | 513.24±295.13 | 499.35±208.33 | 0.894 |
| Alpha RP | 0.44±0.11 | 0.48±0.16 | 0.513 | 0.42±0.11 | 0.42±0.15 | 0.960 |
| Theta AP | 643.18±350.98 | 691.21±397.16 | 0.759 | 228.69±121.80 | 260.60±173.61 | 0.614 |
| Theta RP | 0.17±0.06 | 0.18±0.09 | 0.859 | 0.20±0.06 | 0.21±0.10 | 0.594 |
| Delta AP | 1019.09±573.53 | 894.60±308.86 | 0.506 | 303.31±129.71 | 290.86±114.12 | 0.805 |
| Delta RP | 0.27±0.08 | 0.24±0.11 | 0.445 | 0.27±0.08 | 0.26±0.11 | 0.782 |
| AP, uV^2^. | | | | | | |

**Table 8.** The PRI change in the NBO and control group

| High-power EEG | Global area | | | Fronto-central area | | |
| --- | --- | --- | --- | --- | --- | --- |
| Pre-intervention | NBO group | Control group | p-value | NBO group | Control group | p-value |
| TAR | 0.19(0.14, 0.32) | 0.21(0.14, 0.32) | 0.978 | 0.21(0.18, 0.33) | 0.26(0.21, 0.32) | 0.567 |
| DAR | 0.25(0.18, 0.34) | 0.30(0.17, 0.36) | 0.978 | 0.22(0.15, 0.39) | 0.24(0.17, 0.35) | 0.723 |
| DTABR | 0.42(0.30, 0.63) | 0.40(0.31, 0.59) | 0.978 | 0.38(0.31, 0.62) | 0.43(0.32, 0.63) | 0.683 |
| FOAR | 0.93(0.71, 1.36) | 0.73(0.48, 1.14) | 0.177 | NA | NA | NA |
| Post-intervention |  |  |  |  |  |  |
| TAR | 0.17(0.12, 0.33) | 0.23(0.15, 0.38) | 0.605 | 0.24(0.15, 0.32) | 0.31(0.20, 0.40) | 0.261 |
| DAR | 0.24(0.17, 0.32) | 0.23(0.19, 0.34) | 0.892 | 0.22(0.15, 0.28) | 0.32(0.18, 0.36) | 0.461 |
| DTABR | 0.39(0.27, 0.60) | 0.39(0.30, 0.63) | 0.643 | 0.42(0.26, 0.63) | 0.50(0.39, 0.62) | 0.428 |
| FOAR | 0.73(0.58, 1.21) | 0.84(0.71, 1.04) | 0.495 | NA | NA | NA |
| Beta AP reduction rate | 0.04(-0.06, 0.19) | -0.13(-0.20, 0.01) | 0.023 | 0.03(-0.10, 0.19) | -0.18(-0.27, -0.08) | 0.002 |
| Alpha AP reduction rate | 0.03(-0.08, 0.21) | 0.01(-0.16, 0.12) | 0.723 | 0.04(-0.06, 0.26) | -0.01(-0.18, 0.09) | 0.047 |
| Theta AP reduction rate | 0.12(-0.07, 0.38) | -0.12(-0.23, -0.01) | 0.026 | 0.06(-0.07, 0.38) | -0.19(-0.37, -0.05) | 0.016 |
| Delta AP reduction rate | 0.13(-0.16, 0.34) | 0.05(-0.13, 0.19) | 0.397 | 0.16(0.07, 0.35) | -0.05(-0.14, 0.10) | 0.012 |
| Normal-power EEG |  |  |  |  |  |  |
| Pre-intervention |  |  |  |  |  |  |
| TAR | 0.47(0.32, 0.70) | 0.33(0.21, 0.65) | 0.361 | 0.60(0.47, 0.85) | 0.48(0.26, 1.10) | 0.608 |
| DAR | 0.94(0.48, 1.21) | 0.43(0.31, 0.72) | 0.082 | 1.04(0.62, 1.31) | 0.59(0.40, 0.97) | 0.150 |
| DTABR | 1.06(0.72, 1.55) | 0.67(0.47, 1.03) | 0.134 | 1.00(0.92, 1.72) | 0.87(0.51, 1.40) | 0.277 |
| FOAR | 0.85 (0.44, 1.05) | 0.50 (0.23, 0.66) | 0.087 | NA | NA | NA |
| Post-intervention |  |  |  |  |  |  |
| TAR | 0.37(0.33, 0.67) | 0.38(0.20, 0.71) | 0.776 | 0.44(0.41, 0.76) | 0.48(0.25, 0.97) | 0.776 |
| DAR | 0.72(0.46, 0.80) | 0.49(0.27, 0.80) | 0.277 | 0.79(0.47, 0.99) | 0.60(0.32, 1.08) | 0.820 |
| DTABR | 0.79(0.68, 1.12) | 0.76(0.40, 1.32) | 0.531 | 0.89(0.73, 1.22) | 0.93(0.48, 1.49) | 0.820 |
| FOAR | 0.76 (0.53, 1.09) | 0.53 (0.25, 0.89) | 0.077 | NA | NA | NA |
| Beta AP reduction rate | 0.08(0.00, 0.13) | 0.00(-0.06, 0.04) | 0.026 | 0.13(-0.01, 0.16) | -0.01(-0.09, 0.04) | 0.012 |
| Alpha AP reduction rate | -0.03(0.14, 0.23) | -0.03(-0.27, 0.06) | 0.469 | -0.11(-0.23, 0.19) | -0.18(-0.30, 0.01) | 0.369 |
| Theta AP reduction rate | 0.07(-0.03, 0.34) | -0.09(-0.21, 0.06) | 0.034 | 0.15(-0.06, 0.36) | -0.10(-0.24, 0.05) | 0.034 |
| Delta AP reduction rate | 0.35(-0.26, 0.47) | -0.05(-0.13, 0.11) | 0.099 | 0.30(0.01, 0.49) | 0.03(-0.21, 0.15) | 0.060 |
| NA: no available. | | | | | | |

**Table 9.** The wavelet entropy change in the NBO and control group

| High-power EEG | NBO group | Control group | p-value |
| --- | --- | --- | --- |
| Global area |  |  |  |
| Pre-intervention | 33.97±2.27 | 34.62±2.20 | 0.482 |
| Post-intervention | 34.20±1.64 | 34.16±2.53 | 0.966 |
| △entropy | 0.23±1.43 | -0.46±1.26 | 0.229 |
| Fronto-central area |  |  |  |
| Pre-intervention | 9.64±0.76 | 9.95±0.64 | 0.304 |
| Post-intervention | 9.84±0.46 | 9.78±0.82 | 0.844 |
| △entropy | 0.20±0.53 | -0.16±0.51 | 0.107 |
| Normal-power EEG |  |  |  |
| Global area |  |  |  |
| Pre-intervention | 36.16±2.13 | 35.83±2.69 | 0.742 |
| Post-intervention | 36.76±2.31 | 36.22±2.57 | 0.598 |
| △entropy | 0.59±1.65 | 0.39±0.98 | 0.711 |
| Fronto-central area |  |  |  |
| Pre-intervention | 10.50±0.64 | 10.38±0.88 | 0.715 |
| Post-intervention | 10.59±0.67 | 10.52±0.77 | 0.814 |
| △entropy | 0.09±0.57 | 0.14±0.29 | 0.794 |
| △entropy: pre-intervention minus post-intervention entropy. | | | |

**Table 10.** The spectral power and PRI change after long-term NBO performance in the high-power EEG

| High-power EEG | Global area | | | Fronto-central area | | |
| --- | --- | --- | --- | --- | --- | --- |
|  | Pre-intervention | Post-intervention | p-value | Pre-intervention | Post-intervention | p-value |
| Beta AP | 681.12±187.11 | 649.03±369.82 | 0.783 | 238.03±90.73 | 215.37±120.95 | 0.503 |
| Alpha AP | 4932.97±1087.62 | 3354.23±1615.49 | 0.030 | 1724.06±533.09 | 1118.20±530.81 | 0.041 |
| Theta AP | 831.03±545.09 | 574.57±351.43 | 0.090 | 319.01±249.66 | 223.97±165.39 | 0.110 |
| Delta AP | 1282.63±230.07 | 936.46±405.73 | 0.098 | 387.98±133.58 | 307.16±173.10 | 0.206 |
| Beta RP | 0.09±0.02 | 0.12±0.06 | 0.095 | 0.09±0.03 | 0.12±0.05 | 0.071 |
| Alpha RP | 0.64±0.08 | 0.60±0.12 | 0.116 | 0.65±0.13 | 0.60±0.15 | 0.022 |
| Theta RP | 0.11±0.06 | 0.10±0.04 | 0.922 | 0.11±0.08 | 0.12±0.06 | 0.819 |
| Delta RP | 0.17±0.02 | 0.17±0.04 | 0.788 | 0.15±0.03 | 0.16±0.06 | 0.247 |
| TAR | 0.16(0.08, 0.28) | 0.17(0.09, 0.31) | 0.225 | 0.14(0.08, 0.36) | 0.19(0.10, 0.38) | 0.043 |
| DAR | 0.27(0.21, 0.32) | 0.29(0.20, 0.43) | 0.893 | 0.18(0.16, 0.36) | 0.28(0.17, 0.49) | 0.080 |
| DTABR | 0.35(0.28, 0.51) | 0.36(0.29, 0.52) | 0.500 | 0.28(0.22, 0.58) | 0.35(0.27, 0.60 | 0.225 |
| FOAR | 0.54(0.43, 0.89) | 0.75(0.55, 1.11) | 0.138 | NA | NA |  |
| NA: no available. | | | | | | |


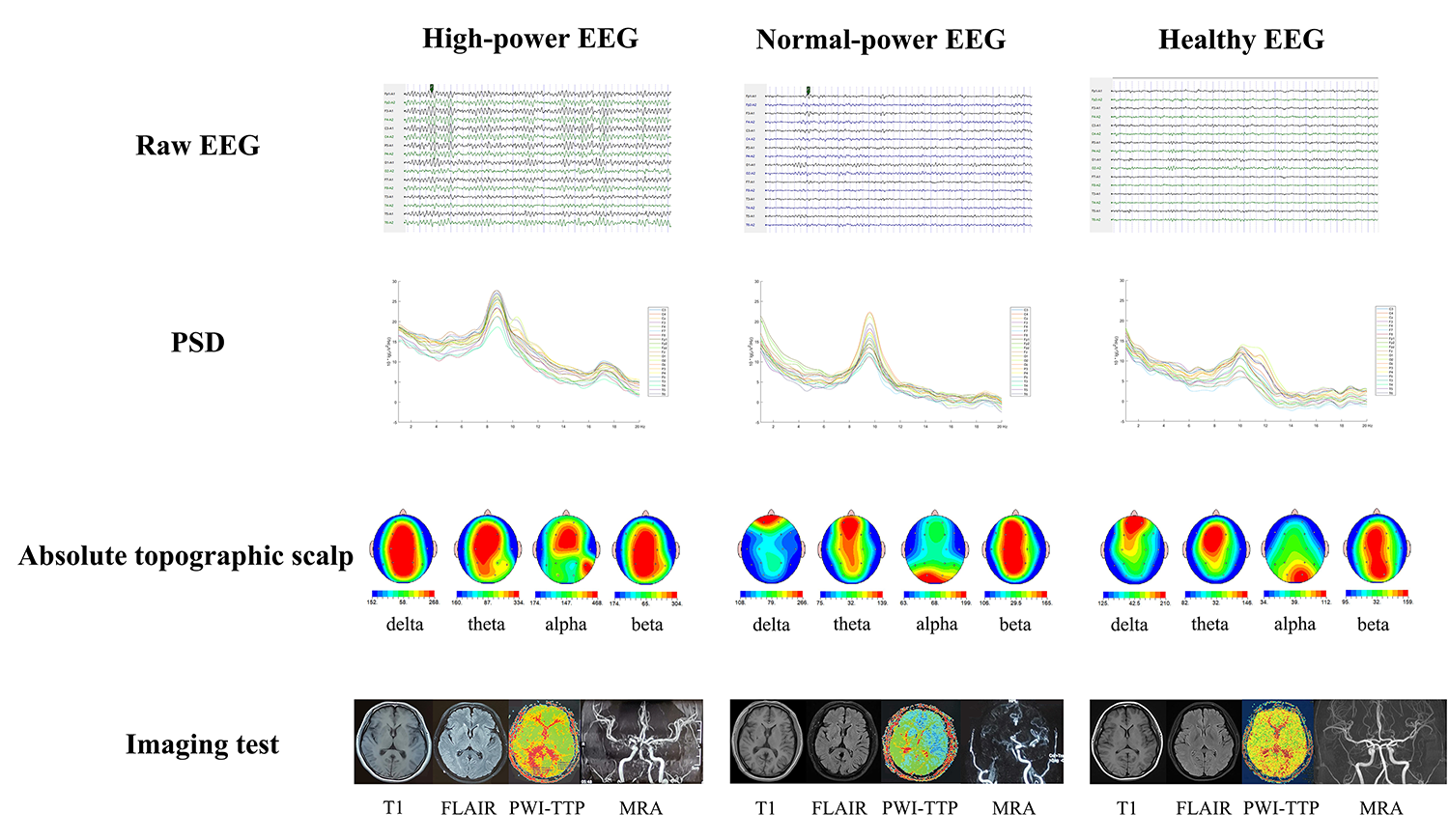


**Figure 1S.** An example of the high-power EEG, the normal-power EEG and the healthy EEG. (1) The high-power EEG is from a 51-year female with right middle cerebral artery and basilar artery severe stenosis, and right middle cerebral artery moderate stenosis. The raw EEG map shows the abnormal high voltage and amplitude oscillations over the range 1-20Hz, especially for the fronto-central electrodes. The PSD shows extremely high alpha power density. Absolute topographic scalp presents all of the delta, theta, alpha and beta frequencies had high power surrounding fronto-centro-parietal electrode area. (2) The normal-power EEG is from a 62-year male with right internal carotid artery and left middle cerebral artery severe stenosis. The raw EEG map shows paroxysmal theta activity release under the normal-voltage EEG background. There is no extremely high power density of the each frequency in the PSD diagram. Absolute topographic scalp shows normal power over the global electrodes. (3) The healthy EEG is from 36-year female. The oscillation power at raw EEG map is substantially lower than the patient with high-power EEG, and less theta frequency releases compared with the patient with normal-power EEG. The PSD is lower than the high-power EEG and normal-power EEG, and less slow wave activities are shown in the absolute topographic scalp.

**
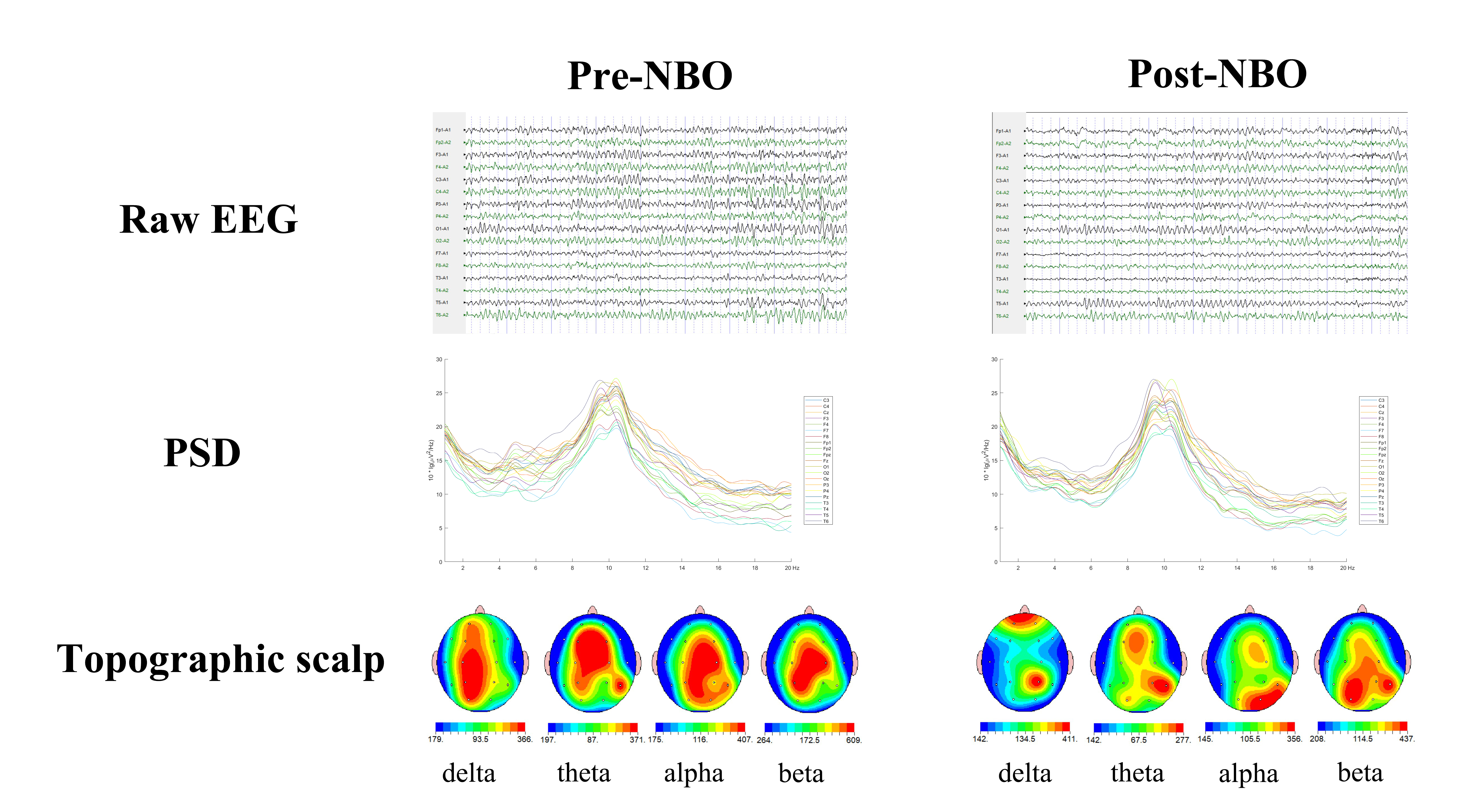
**

**Figure 2S.** EEG changes prior to and post-NBO administration. A 71-year female with bilateral MCA and ACA severe stenosis is performed with NBO between the two EEG recordings. The abnormal high-voltage oscillations in the raw EEG maps are suppressed after NBO therapy. As for PSD diagram, the slow frequencies power density is also restrained after NBO performance. Meanwhile, the topographic scalp also shows the high power is ameliorated after undergoing NBO therapy.
